# Identification of Relevant Phytochemical Constituents for Characterization and Authentication of Tomatoes by General Linear Model Linked to Automatic Interaction Detection (GLM-AID) and Artificial Neural Network Models (ANNs)

**DOI:** 10.1371/journal.pone.0128566

**Published:** 2015-06-15

**Authors:** Marcos Hernández Suárez, Gonzalo Astray Dopazo, Dina Larios López, Francisco Espinosa

**Affiliations:** 1 Aula Dei Scientific Technological Park Foundation, Zaragoza, Spain; 2 Department of Geological Sciences, College of Arts and Sciences, Ohio University, Athens, United States of America; 3 Department of Physical Chemistry, Faculty of Science, University of Vigo, Ourense, Spain; 4 Department of Plant Physiology, Ecology and Earth Sciences, Faculty of Science, Extremadura University, Badajoz, Spain; University of Tsukuba, JAPAN

## Abstract

There are a large number of tomato cultivars with a wide range of morphological, chemical, nutritional and sensorial characteristics. Many factors are known to affect the nutrient content of tomato cultivars. A complete understanding of the effect of these factors would require an exhaustive experimental design, multidisciplinary scientific approach and a suitable statistical method. Some multivariate analytical techniques such as Principal Component Analysis (PCA) or Factor Analysis (FA) have been widely applied in order to search for patterns in the behaviour and reduce the dimensionality of a data set by a new set of uncorrelated latent variables. However, in some cases it is not useful to replace the original variables with these latent variables. In this study, Automatic Interaction Detection (AID) algorithm and Artificial Neural Network (ANN) models were applied as alternative to the PCA, AF and other multivariate analytical techniques in order to identify the relevant phytochemical constituents for characterization and authentication of tomatoes. To prove the feasibility of AID algorithm and ANN models to achieve the purpose of this study, both methods were applied on a data set with twenty five chemical parameters analysed on 167 tomato samples from Tenerife (Spain). Each tomato sample was defined by three factors: cultivar, agricultural practice and harvest date. General Linear Model linked to AID (GLM-AID) tree-structured was organized into 3 levels according to the number of factors. p-Coumaric acid was the compound the allowed to distinguish the tomato samples according to the day of harvest. More than one chemical parameter was necessary to distinguish among different agricultural practices and among the tomato cultivars. Several ANN models, with 25 and 10 input variables, for the prediction of cultivar, agricultural practice and harvest date, were developed. Finally, the models with 10 input variables were chosen with fit’s goodness between 44 and 100%. The lowest fits were for the cultivar classification, this low percentage suggests that other kind of chemical parameter should be used to identify tomato cultivars.

## Introduction

Wild tomatoes are native from western South America. The generic status of wild tomatoes within the family of Solanaceae has been a matter of controversy since the eighteen century. Linnaeus in 1753 classified tomatoes in Solanum genus while Miller, a contemporary of Linnaeus, classified tomatoes in a genus Lycopersicon. At present, tomato is classified as *Solanum lycopersicum* cv Mill. There are a large number of tomato cultivars with a wide range of morphological, chemical, nutritional and sensorial characteristics [1].

Tomato is one of the most widely consumed fresh vegetables in the industrialized world. It is also widely used by the food industries as raw material for the production of purees, ketchup and other products. Tomato is considered as a functional food due to its special composition of bioactive compounds, as it is a good source of minerals, fibre, vitamins and antioxidants such as lycopene. Tomato is also the most common vegetable in the Mediterranean diet, a diet known to have health benefits, especially to avoid the development of chronic degenerative diseases [2].

However, many factors are known to affect the nutrient content of tomatoes, such as cultivar, climate, geography, soil and water geochemistry and agricultural practices [3]. This explains the quite large number of studies aiming to evaluate and improve the quality of tomato fruit. The obstacle has been, however, that the interactions between genetic properties, environmental and agricultural practices are complicated. A complete understanding of the effect of these factors would require not just an exhaustive experimental design, but also a multidisciplinary scientific approach and a suitable statistical method to search for patterns in the behaviour of the variables investigated [4].

Although sensory evaluation is the best method to characterize tomato fruit, these test are expensive, time-consuming, and require a panel with a considerable number of experts, and panellists often constitute the first source of variation. Thus, when a high number of samples are to be analysed, this type of evaluation can be substituted by the multivariate analytical techniques to discover hidden relationships, correlations, trends and associations in data [5].

However, there are considerable difficulties in analysing and interpreting this kind of data so it is necessary to apply statistical tools that can reveal behaviour patterns. Some multivariate analytical techniques such as Principal Component Analysis (PCA), Factor Analysis (FA), Linear Discriminate Analysis (LDA) and Cluster Analysis (CA) have been widely applied to this problem. PCA reduces the dimensionality of a data set having a large number of inter-correlated variables, while retaining as much as possible the information present in the original data. The reduction is achieved through a linear transformation to a new set of uncorrelated latent variables that express most of the variation of the original variables. FA transforms a n-dimensional data structure to another with considerably less dimensions, like PCA, but gives the opportunity to the researcher to select between uncorrelated factors [6].

CA is one of the most useful statistical tools used in chemometrics for discovering groups and localizing (identifying) interesting distributions and patterns in the underlying information contained in the data. LDA is based on the extraction of discriminant functions of the independent variables by means of a qualitative dependent variable and several quantitative independent variables. The method supplies a number of linear discriminant functions to provide a method for predicting the group into which a new case will most likely fall [7].

Although some of these methods are clearly better than others under a given set of circumstances, there is no single ‘‘best” approach, but in some cases it is not useful to replace the original variables with these linear combinations. In multienvironment trials, biplot analysis is being increasingly used in the analysis of this kind of data. The biplot methods proposed by Gabriel [8] are a graphical display of multivariate data in two dimensions. This is done by representing the variables as vectors in the same plane and the correlations between them as the angles between those vectors. Also, tree-structures and other models based on Artificial Intelligent (A.I.), such as the Artificial Neural Networks (ANNs) must be taken account.

Trees-structures are used for the classification, least squares regression and analysis of censored survival data [9]. The first tree-structure was the Automatic Interaction Detection (AID). AID algorithm is based on the partitioning of a group into other subgroups according to an independent variable called predictor linked to a dependent or response variable able to distinguish the subgroups inside the original group. In each subgroup, there are new individuals that can be affected by other variables. In this way, AID detects the automatic interaction among individuals and variables [10].

Regarding ANNs, they have been applied in many fields such as flow river prediction to prevent floods [11], to predict the average monthly wind speed in one station from others neighbouring stations [12], to modelling and control of nonlinear systems [13], to predict the traffic flows in an urban street [14], to predict the critical micelle concentration (CMC) values in different surfactants [15], or even in predictive systems on the stock market [16]. Neural Networks are a modeling method that imitates the human brain [17]. The basic processing unit in an artificial neural network (neuron) is based on the biological cell, thus an ANN will have a large number of interconnected neurons. In this simulation method, the database is divided into training and validation data. The large number of connections make the ANN capable of finding the more important relationships between the variables (key relationships) in the training database, used to generate the model, and then apply that knowledge to new cases previously unseen, known as validation data [18]. Thus, ANN do not look for the formulation of a physical or chemical law in the training database used for model implementation, but rather, they look for a relationship among the data to achieve a result close to the expected value. This modelling method is particularly useful for complex problems where there are many variables involved and our knowledge of the variability of these variables and their interactions is limited.

In this paper, two objectives were considered. The first one was to identify those relevant phytochemical constituents responsible for the main differences among tomatoes samples by Automatic Interaction Detection method and the second objective was to develop an authentication Artificial Intelligent model that could predict with accuracy the cultivar, the production type and the harvest date.

The reasons for selecting the AID algorithm as a method to identify the relevant phytochemical constituents are as follows: AID algorithm uses original variable instead of latent variables used in CPA and AF, and trees-structures are easily understandable and interpretable. A correct identification of the relevant phytochemical parameters allows to understand the relationship between environment and chemical composition. This understanding could help genetic improvement programs.

Some authors have pointed out that CA and LDA frequently fail to differentiate food samples because linear functions are probably not appropriate to describe this kind of data. There are many external factors that can have influence in the chemical composition [7]. Thus, the use of ANN as a mathematical tool is feasible because ANNs are based on non-linear functions. An adequate model allows to develop a method to detect food fraud.

## Materials and Methods

### Tomato sampling and sample preparation

One kg samples were collected during four different harvesting periods (October, December, February and April), but at the same degree of ripeness according to the Dutch “kleurstadia” tomato-colour scale. They belonged to five cultivars (Dorothy, Boludo, Dominique, Thomas and Dunkan) grown under three farming practices: conventional, organic, and no-soil on coconut fibre substrate. In the trials, the UNE 155102 standard for the controlled production of tomatoes and several European regulations on organic production and on maximum residue levels of pesticides were taken into account. Regarding the no soil tomatoes, the nutrient solution consisted of 12mM N-NO_3_^-^, 0.5 mM N-NH_4_^+^, 1.6 mM P-H_2_PO_4_, 7 mM K^+^, 4.5 mM Ca^2+^, 2 mM S-SO_4_^2-^, 5 μM FeEDTA, 2 μM MnS0_4_, 1 μM ZnSO_4_, 0.25 μM CuSO_4_, 0.1 μM Na_2_MoO_4_, and 50 μM H_3_BO_3_. pH 5.5–6 and EC 2.5–2.8 dS m^-1^. A total of 167 samples tomato samples were provided by ACETO Company (Asociación Provincial de Cosecheros Exportadores de Tomates de Tenerife, Spain) which has the trial field in the South of Tenerife. The recolection does not require specific permission and the field studies did not involve endangered species. The samples are described in Hernández et al. [19].

### Sample preparation method

Three tomatoes were randomly selected from each tomato sample for analysis. The samples were hand-rinsed with ultra-pure water, shaken to remove any excess water, and gently blotted with a paper towel. They were then mixed and homogenized to homogeneous puree. A fraction of this purée was desiccated, homogenized again, and stored in a polyethylene tube (10 mL) at room temperature until assay for metals, protein and total fibre. The rest was stored in a polyethylene tube (15 mL) at -80°C for the measurement of the other chemical parameters: fructose, glucose, organic acids (citric, malic, oxalic, pyruvic, fumaric and ascorbic), lycopene, phenolic compounds and hydroxycinnamic acids (caffeic, *p*-coumaric, chlorogenic and ferulic). Data are expressed as % or quantities per fresh weight.

### Analytical parameters

The mineral concentration was determined by atomic absorption spectrophotometry following nitric acid digestion except for phosphorus which was measured by a colorimetric method, using a vanadate-molybdate reagent. The nitrogen concentration was determined by the Kjeldahl method, and then the protein concentration calculated using a nitrogen factor of 6.25 [20]. The ascorbic acid and total fibre content were determined using approved methods described by AOAC [20]. Analytical HPLC methods were used to measure the concentrations of sugars (glucose and fructose), organic acids (citric, malic, oxalic, pyruvic and fumaric acids) and hydroxycinnamic acids (chlorogenic, caffeic, p-coumaric, and ferulic acids). The analytical HPLC methods used were the same previously described in Hernández et al. [19]. The chemical parameters were determined in triplicate for each sample. Supplementary file shows the mean values according to the harvest date (S1 Table), agricultural practices (S2 Table) and tomato cultivars (S3 Table).

### Statistics

#### Automatic Interaction Detection (AID) analysis

According to Santesmeses [21], AID analysis is a sequentially repeated one-way ANOVA. In each step, the algorithm reveals the best variable able to divide the initial group. The partition among categories must maximize the inter-groups variance and minimize intra-group variance. However, the data of this study are affected by three factors (harvest date, agricultural practice and cultivar) so this concept must be adjusted.

In principle, ANOVA can be used with any number of factors. With one factor is called one-way ANOVA and with two or more factors are called factorial ANOVA. The linear model that represents the structure of the experiment is called General Linear Model (GLM). This model contains a term for the baseline, a term for each individual factor or main effect, a term for each interaction, and a term for error. GLM is used to find out how the average value of the dependent variable differs across the categories being compared [22].

The requirements to apply GLM are the same that ANOVA, normal distribution and homoscedasticity. Both requirements were achieved in this study by means of the standardization according to Eq 1:

$$Z_{ij}=\frac{x_{ij}-\;{\bar{x}}_{j}}{\sigma_{j}}{\text{z}}_{\text{ij}}=\frac{{\text{X}}_{\text{ij}}-{\bar{\text{X}}}_{\text{j}}}{\sigma_{\text{j}}}$$ (1)

Where *X*_ij_ is each data point within the variable j, ${\bar{X}}_{j}$ is the average of the variable *j* and *σ*_j_ is the standard deviation of the variable *j*. The Eq 1 transforms the data set to have zero mean and unit varianza. This transformation also allows to give each variable equal weight in the statistical analysis [7].

The GLM equation adapted to our case was (Eq 2):

$$\bar{y}{\;}_{ijk}=\;\mu +\;c_{i}+\;p_{j}+\;h_{k}+\;c_{i}\;\cdot \;p_{j}+\;c_{i}\;\cdot \;h_{k}+\;p_{j}\;\cdot \;h_{k}+\;c_{i}\;\cdot \;p_{j}\;\cdot \;h_{k}+\;\varepsilon_{ijk}$$ (2)

Where ${\bar{y}}_{ijk}$ is the mean value of the response variable of the *i* ^*th*^ tomato cultivar (Dorothy, Boludo, Dominique, Thomas and Dunkan), *j* ^th^ production system (conventional, organic and no-soil), *k* ^*th*^ harvest date (October, December, February, April), *μ* is the baseline, c_*i*_ is the cultivar effect, *p*_j_ is the production system effect, *h*_*k*_ is the harvest date effect and ε_*ijk*_ is the error associated to the model.

The following criterion were considered to obtain the possible solutions for selecting a predictor that can divide each group and can produce a tree-structure: i) the response variable (the chemical parameter) must depend on one predictor to be considered stable and suitable to organize the results as a tree-structure, ii) the *p*-value of the adjusted model must be the lowest, and iii) in case of coincidence, the explained variance of the model must be the highest. Once the predictor was selected, a Bonferroni post hoc test [23] was performed to identify the subgroups. This process is sequentially repeated until a significant predictor is not observed. All subgroups or nodes obtained were grouped like a tree-structure. The GLM-AID tree-structure is based on the main significant differences among the categories of one factor for one attribute, in our case a chemical parameter.

These calculations were performed using the SPSS version 21.0 for Windows software package.

### Artificial Neural Network

**Fig 1:**
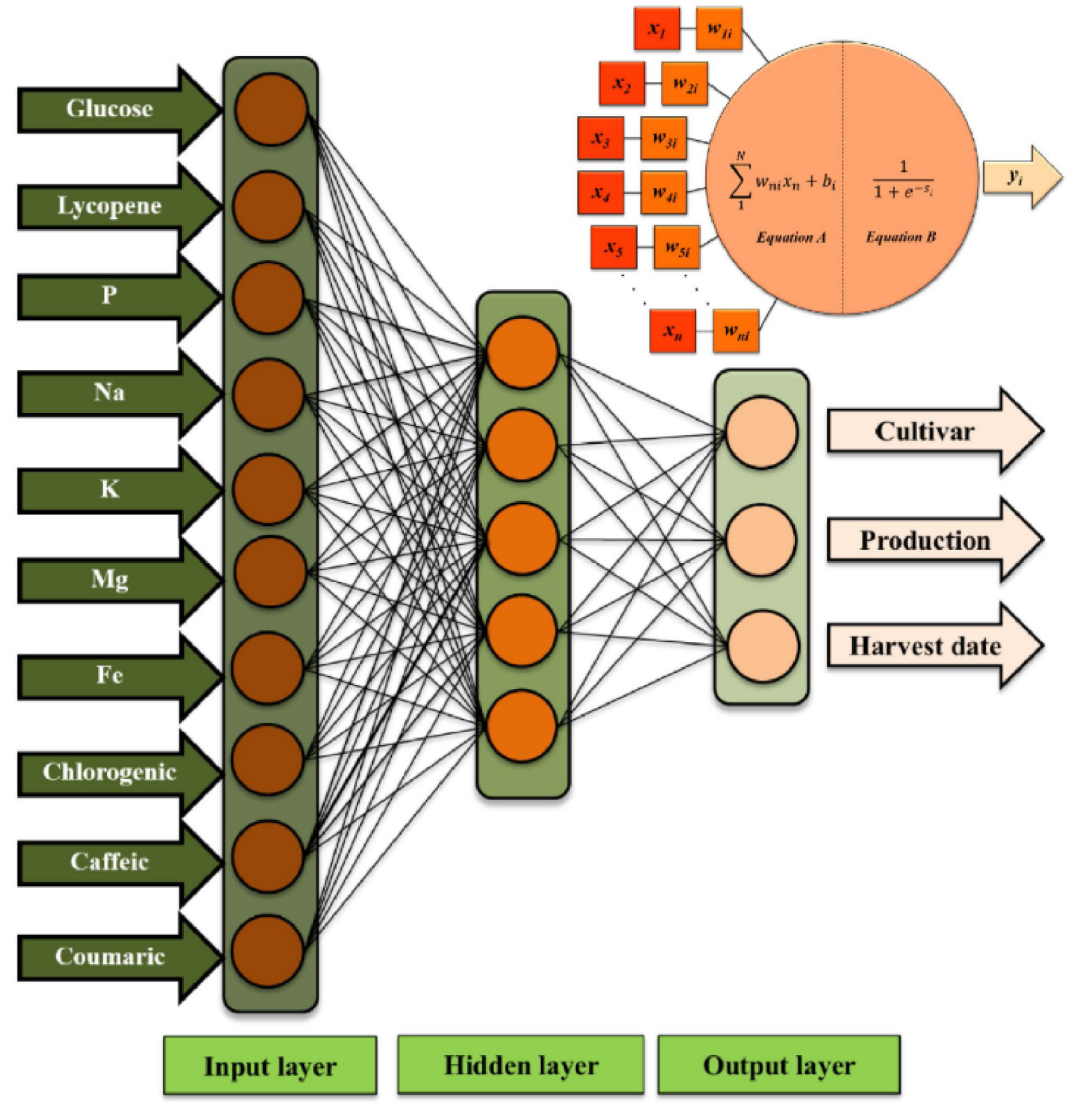
Diagram operational of an artificial neuron and sample diagram for an ANN_3_ model with ten input neurons, five neurons in the intermediate layer and three output neurons, that’s, with a topology of 10-5-3.

The development of a neural network is based on the summation of the operations in each of the neurons that compose the system. The information is entered into the system by a vector *X*_*i*_ = (*X*_1_, *X*_2_, …*X*_*n*_) (Fig 1).

All information in the input vector is processed by a mathematical function that transfers this information to the first intermediate layer. The propagation function commitment is to add all the input data and generate a single response (Fig 1, Equation A). In this equation *N* is the neurons number in the first Neural network layer, denominated input layer, *w*_*ni*_ is the weight (which indicates how important the connection is) between neurons in input layer (*n*) and neurons in intermediate layer (*i*), and finally, *b*_i_ corresponds with the biases associated to the neurons in the intermediate layer (Eq 3).

$$S_{i}=\;\overset{N}{\underset{n-1}{\sum }}w_{ni}x_{n}+\;b_{i}$$ (3)

The values obtained by the propagation function are used by other mathematical function, called activation function (Eq 4), to provide an output value (*y*_*i*_) as a function of the internal state [24] and exceeds a threshold value [25]. Different activation functions can be used but in this work it was used the sigmoidal function (Fig 1, Equation B).

$$y_{i}=\;\frac{1}{1+e^{-S_{i}}}$$ (4)

All information entered in the Neural network is propagated to the output layer, where an output value is generated (*y*_0_). This value is compared with the experimental value (*d*_*0*_), and the error produced by the Neural network (Eq 5) can be calculated.

$$E=\;\frac{1}{2}\overset{0}{\underset{0=1}{\sum }}{\left(d_{0}-y_{0}\right)}^{2}$$ (5)

#### Implementation procedure and choice of the best Artificial Neural Networks

The first step to develop the different Neural Network models is the selection of the data (training data) that we use to train the system (training phase) and then the data (validation data) for check the prediction power of different Neural Network models (validation phase). As discussed in the previous section, there are 167 tomato samples analysed, 151 had been chosen to be part of the training phase and 16 were reserved for the validation phase and to check the prediction power of different Neural Networks implemented.

In this work we have implemented a high number of Neural Networks to achieve the desired values of cultivar, production type and harvest date. Once all Neural Networks have been developed, we need to check the good predictive power for training and validation phase. Traditionally, the predictive power is calculated by different statistical parameters such as; i) the Root Mean Square Error (RMSE) comparing predicted values and real values, ii) the Individual Percentage Deviations (IPD) or iii) the Average Percentage Deviations (APD). These type of calculations are made when the output variables are continuous, however, in this paper we are working with discrete variables that can only take a specific value. For this reason we have calculated the predictive power of different Neural Networks as a function of the percentage of success (match between the predicted and the real variables: Harvest date, Production and Cultivar), such as the Average Percentage of Success (APS) (Eq 6).

$$APS=\;\frac{\left(\sum_{i=1}^{N}Success\right)100}{N}$$ (6)

For the implementation of different ANN models we used EasyNN plus, Version 14.0d, by Neural Planner Software. The program was installed in two different computing equipment; i) a Personal Server with an Intel Core i7 processor with RAM memory of 8 GB, and ii) a Personal Server with an Intel Core i5 processor with RAM memory of 4 GB, both servers with virtual machines.

#### Notation for the developed models

To clearly identify the different topologies of ANN developed, we used the following notation that considers each neuron in the different layers of the neural network model.

$$N_{input\;layer}-\;N_{intermediate\;layer}-\;N_{output\;layer}$$ (7)

Where *N*_*input layer*_ and *N*_*output layer*_ represents the neurons in the input and output layer, that’s the neurons that receive information from the outside, and the neurons that generate output to the input information. *N*_*intermediate layer*_ corresponds with the neurons in the intermediate layer.

#### Input variables reduction for simple models (ANN_3_ and ANN_4_)

To reduce personal costs, material and analytical cost, and time, we have reduced the input variables of the first models (models ANN_1_ and ANN_2_ with 25 input variables) based on the importance of all input variables. The importance was determined by the sum of absolute values of all weights between the input neuron and all intermediate neurons. The new 10 input variables for simple models ANN_3_ and ANN_4_ were; Glucose, Lycopene, P, Na, K, Mg, Fe, cholorogenic acid, caffeic acid and *p*-coumaric acid. The results of ANNs model are provided as supplementary material (S4 Table).

## Results and Discussion

### General composition of tomatoes and influential factors

The average chemical composition of the tomato samples and the factors that significantly influence this composition are shown in Table 1. The mean contents of fructose (1.28±0.41%) and glucose (1.29±0.40%) were similar and within the range of concentration found by Cebolla-Cornejo et al. [5]. According to the GLM results, the mean glucose content depends on more factors than fructose. The fructose content only depends on agricultural practice and date of harvest. In comparison, Gautier et al. [26] observed that light and temperature had no significant effects on final sugar content.

**Table 1:** Mean content of the chemical parameters and estimation of the influence factors (*p*-value). Data are expressed as % or quantities per fresh weight.

		*p-Values*							
Parameter	Content	*c*_*i*_	*h*_*k*_	*p*_*j*_	*c*_*i*_ *·h*_*k*_	*c*_*i*_ *·p*_*j*_	*p*_*j*_ *·h*_*k*_	*c*_*i*_ *·p*_*j*_ *·h*_*k*_	Explained variance (%)^a^
Fructose (%)	1.28±0.41		0.012^1^	0.001				0.030	47.6
Glucose (%)	1.29±0.41		0.000	0.000	0.004	0.033			56.7
Total fiber (%)	1.81±0.56		0.006	0.008	0.001	0.027	0.000	0.003	57.3
Protein (%)	0.80±0.15		0.005	0.018					36.2
Phenolic compound (mg/100g)^b^	20.41±4.3		0.020		0.005				38.3
Lycopene (mg/ 100g)	2.31±0.72		0.000						53.9
P (mg/kg)	246±61			0.000				0.034	50.3
Na (mg/kg)	92.4±63.4	0.004		0.000		0.004	0.018		58.8
K (mg/kg)	2522±512		0.000	0.015					52.2
Ca (mg/kg)	67.5±18.6	0.010	0.001	0.000	0.002	0.000	0.009		59.3
Mg (mg/kg)	115±22		0.000	0.000				0.038	59.3
Fe (mg/kg)	1.92±0.05	0.000	0.020		0.040	0.002	0.000		53.3
Cu (mg/kg)	0.30±0.15		0.000	0.022	0.017				50.4
Zn (mg/kg)	0.77±0.21		0.045	0.000		0.024			49.9
Mn (mg/kg)	0.60±0.21	0.000	0.008	0.000		0.000	0.032		69.8
Ascorbic acid (mg/ 100g)	15.3±4.48					0.035			37.8
Oxalic acid (mg/ 100g)	25.6±9.3			0.011					37.5
Pyruvic acid (mg/ 100g)	1.37±0.77	0.031	0.001		0.000	0.028		0.004	59.8
Malic acid (mg/ 100g)	78.3±40.2	0.000	0.000	0.000	0.001	0.000	0.002	0.000	75.0
Citric acid (mg/ 100g)	354±121		0.000	0.043	0.037	0.013			48.2
Fumaric acid (mg/ 100g)	2.77±1.22		0.009						36.0
Chlorogenic acid (mg/ 100g)	0.59±0.05		0.000		0.027		0.030		50.7
Caffeic acid (mg/ 100g)	0.04±0.01		0.000						53.9
Ferulic acid (mg/ 100g)	0.09±0.04	0.032	0.000			0.001	0.022		57.2
*p*-Coumaric acid (mg/ 100g)	0.02±0.03		0.000						73.6

^a^ Values used to select the first predictor for the GLM-AID analysis.

^b^ Expressed as galic acid

The mean content of total fibre in the analysed tomatoes was 1.81±0.56% and its content depends on all factors and their interactions (Table 1). Claye et al. [27] observed that the tomato fibre was composed of 87% insoluble fibre and 13% soluble fibre. The mean protein content obtained (0.80±0.15) is significantly influenced by harvest date and production system.

The mean content of phenolic compounds (20.41±4.37 mg galic acid/100g) and lycopene (2.31±0.72 mg/100 g) were similar to those concentrations reported by Slimestad and Verheul [28]. The mean content of lycopene is more significantly influenced by the collection date while the phenolic compounds vary according to the harvest date and the interaction between cultivar and harvest date. These results agree with those of Tedeschi et al. [29].

The individual mineral content of the tomato samples (Table 1) were similar to those concentration found by Tedeschi et al. [29] and Max et al. [30], except for Na (92.4±63.4 mg/Kg) that was highest. The high Na concentration in Tenerife soil, which is derived from alkaline volcanic rocks, the high salinity of the water used in the irrigation, and the influence of the marine aerosol could explain the relatively high concentration of Na in the tomatoes [31]. In Table 1, one can see that the mean content of Na varies mainly with production system (the lowest *p*-value). Our results for Ca, Fe, Zn, Mn and Cu were near to those found by Gundersen et al. [32]. The main significant differences (the lowest *p*-value) are linked to the agricultural practices and collection date, except for the Fe, which is more significantly influenced by the tomato cultivar and the interaction between the production system and the harvest date. Regarding the role of minerals in determining tomato fruit quality, Fanasca et al. [33] suggest that new trials are required to understand the interaction between every element and the fruit tomato quality.

Citric acid (354±121 mg/100 g) was the major organic acid followed by malic (78.3±40.2 mg/100 g) and oxalic (25.6±9.3 mg/100 g). Citric acid is the main agent responsible for the acidity of tomatoes and its concentration was similar to values found by Cebolla-Cornejo et al. [5]. However, the concentration of malic acid of our tomato samples were lower than values found by these authors. Malic acid plays a key role as an important indicator of the freshness of fruits. Oxalic acid forms insoluble salts with calcium and other essential divalent cations producing a decrease in the bioavailability of these nutrients [34]. Oxalic acid and fumaric acid depend on individual factors, agricultural practices and collection date, respectively, while the mean value of ascorbic acid is more significant influenced by the interaction of agro-climatic factors. The mean concentration of ascorbic acid (15.3±4.48 mg/100 g) obtained in this research was very similar to the mean concentrations found by Thybo et al. [35]. The mean content of the rest of the organic acids is more significantly influenced by various factors and their interaction (Table 1).

Four hydroxycinnamic acids, chlorogenic 0.59±0.05 mg/100 g, caffeic 0.04±0.01 mg/100 g, ferulic 0.09±0.04 mg/100 g, and *p*-coumaric 0.02±0.03 mg/100 g were detected in the tomato samples, which agrees with the results by Raffo et al. [36]. In all cases, the collection date had the highest influence on the mean content (Table 1).

### Characterization of the tomato samples

The purpose of GLM-AID is to identify and highlight the main significant differences in order to identify the most relevant phytochemical constituents and characterize the tomato samples. Its results were organized as a tree-structure with 3 levels according to the number of factors (harvest date, agricultural practice and cultivar). In some nodes, new divisions were not possible due to: i) more than one chemical parameter was necessary to distinguish among cases, ii) there were no significant differences among the chemical parameters of the samples inside the node or iii) some cases with only a single sample, the mean values cannot be compared.

The first level of hierarchy was the harvest date. The first chemical parameter selected was the *p*-coumaric acid that divides the samples according to each harvest date. Tomato samples harvested in October (average temperature 22.9±1.2°C, average irradiation 16.9±3.8 W/m) had a mean content of this acid of 0.52±0.23 mg/100 g, 0.17±0.17 mg/100 g in samples collecting in December (average temperature 17.1±0.8°C, average irradiation 12.2±3.4 W/m) and 0.02±0.01 mg/100 g in February samples (average temperature 15.8±1.1°C, average irradiation 14.2±4.7 W/m). However, the *p*-coumaric acid was not detected in the tomato samples collected in April (average temperature 18.5±0.6°C, average irradiation 21.7±4.5 W/m). During this month, the mean value of irradiation was the highest so a clear effect of the climatic conditions on this antioxidant acid was observed. Similar results were obtained by Rosales et al. [37] when they analysed the variations of other antioxidants with climatic condition.

**Fig 2:**
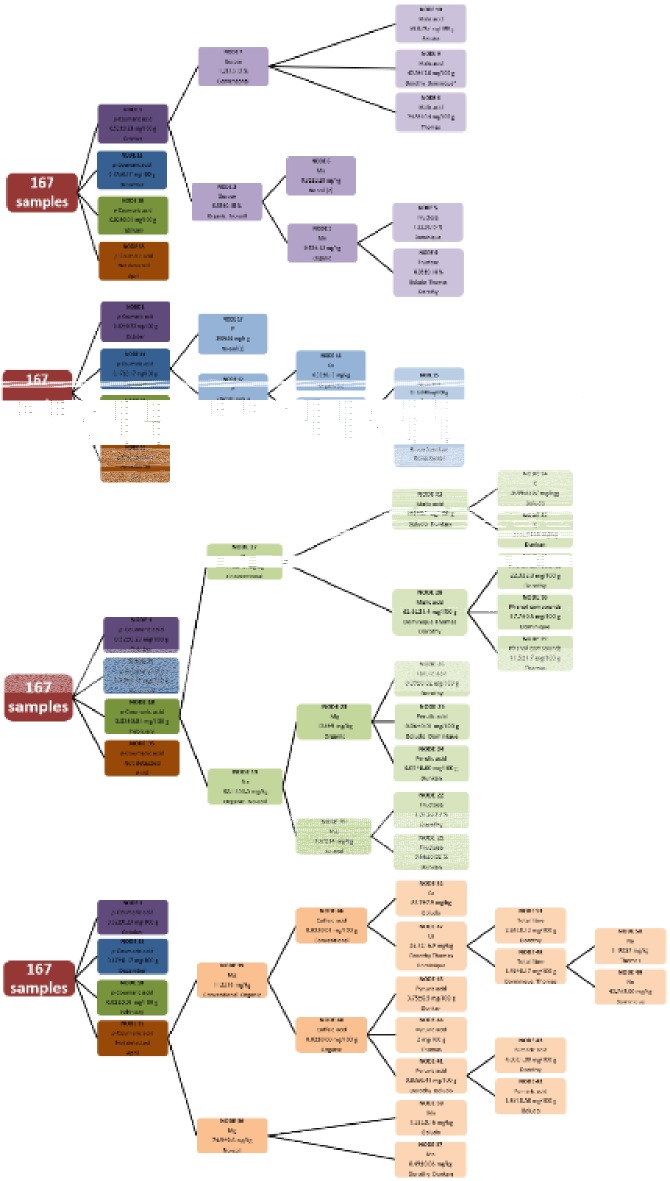
Tree-structured with main significant differences according to the GLM-AID analysis for the tomato samples. 2A October; 2B December; 2C February; 2D April.

More difficulties were observed in analysing a pattern between production methods and chemical composition (2^nd^ level of the tree-structured). The tomato samples collected in October (Fig 2) had the significantly highest concentrations of *p*-coumaric acid (node 1). Regarding agricultural practices, it had significant influence on glucose content. Tomatoes conventionally obtained had the highest values of glucose (node 7, 1.21±0.12) with respect to the other two practices. The malic acid content allows to distinguish the conventional tomato cultivars: Thomas (node 8, 24.8±10.4 mg/100 g), Dorothy (node 9, 42.8±12 mg/100 g) and Boludo (node 10, 59.8±9.7 mg/100 g). Organic and no-soil tomatoes had the lowest glucose content (node 2, 0.85±0.18%). The Mn content allows to distinguish between both practices, 0.62±1.2 mg/kg for organic tomatoes and 0.86±0.2 mg/kg for no-soil tomatoes. Tomato cultivars organically produced were distinguished according to the fructose content (node 4 and 5).

Tomato samples collected in December (Fig 2) had a content close to the average of *p*-coumaric acid (node 11). The content of P and Cu allow to distinguish among agricultural practices. No-soil tomato samples had the significantly highest value of P (node 17, 300±56 mg/kg) in relation to the other two systems (node 12, 226±40 mg/kg). Organic tomatoes had the highest content of Cu (node 16, 0.33±0.13). The Dorothy tomato cultivar had the significantly highest content of ferulic acid (node 15, 0.13±0.03 mg/100 g) while the rest of tomato cultivars showed a similar chemical composition (node 14).

Tomato samples collected in February (Fig 2) had the significantly lowest *p*-coumaric acid content (node 18). Within this group, no-soil tomatoes had the lowest value of Na (node 19, 82.1±53.3 mg/kg) and Mg (node 20, 107±14 mg/100 g) and tomato cultivars belonging to this subgroup differentiate themselves by fructose (nodes 21 and 22). In contrast, organic tomatoes had the highest content of Mg (node 23, 125±9 mg/kg). Tomato cultivars produced by organic practices can be distinguished between them by the content in ferulic acid (nodes 24–26).

Regarding conventional tomato samples, they had the significantly highest content of Na (node 27, 146±72 mg/kg). One can observe two subgroups. The first is formed by the Dominique, Thomas and Dorothy tomato cultivars with the lowest content of malic acid (node 28, 62.6±21.4 mg/100 g) while the second group is formed by the Boludo and Dunkan tomato cultivars which had the highest content of this organic acid (node 32, 152±56 mg/100 g). The phenol content allows to distinguish between Dominique, Thomas and Dorothy (nodes 29–31) while the content of K discriminate between the Boludo and Dunkan tomato cultivars.

*p*-Coumaric acid was not detected in tomato samples collected in April (node 35, Fig 2). One possible negative effect of weather conditions, especially linked to irradiation, may be the cause as discussed above. No-soil tomato samples collected in this month showed the significantly lowest value in Mg (node 36, 74.9±9.6 mg/100g). The Boludo, Dorothy and Dominique tomato cultivars within this subgroup were different according to the Mn content (nodes 37 and 38). Organic and conventional tomatoes had the highest values of Mg (node 39, 112±16 mg/100 g). The difference between both systems was the content in caffeic acid, 0.02±0.00 and 0.03±0.01 mg/100 g respectively. The organic tomato cultivars (nodes 41, 44 and-45) can be distinguished by the content in pyruvic acid. In contrast, the tomato cultivars conventionally obtained were significantly different in the Ca, total fiber and Na content (nodes 47–52).

In order to distinguish tomato samples according to the three agricultural practices, the most significant chemical parameters were glucose, Mn, P, Cu, Na, Mg and caffeic acid. Tomatoes conventionally obtained had the highest mean values of glucose, Na and caffeic acid, while the organic tomatoes had higher concentrations of Cu. No-soil tomato samples had the lowest concentrations of these chemical parameters except for P.

In a previous study HJ-Biplot was used [21]. HJ-Biplot and GLM-AID offer supplementary information. GLM-AID identifies the main chemical parameters responsible for the differences among samples while HJ-Biplot [38] revealed simultaneously the next information: correlation among chemical parameters, similarity among tomato cultivar samples and relationship among tomato samples and chemical parameters. The biplot also showed that when tomatoes have the same degree of ripeness: 1) the climatic conditions may have been more relevant to distinguishing these two groups than the kind of agricultural practice, 2) sugars, organic acids, protein, Mg, and Na were strongly correlated with similar profiles for the conventional and organic samples, 3) with good management practices the type of agricultural practice had little effect on the chemical composition to distinguish between conventional and organic tomatoes, 4) antioxidant compounds are mainly contained in conventional and organic tomato samples harvested in October and 5) unlike the previous case, the kind of agricultural practice seems to have more effect on mineral concentration than the climatic conditions.

### Selection of nutritional markers to authenticate the tomato samples by artificial intelligent models

As mentioned in the previous section, the development of an ANN requires the implementation of many models, using a trial and error method, to obtain the best Neural Networks model to determine the output variables. We implemented over one thousand Neural Networks with different number of input variables, with different topologies (varying the number of neurons in input and output layer) or with different training cycles (to avoid over fitting of the Neural network) to determine the best Artificial Neural Networks to predict the cultivar, production type and harvest date of a tomato. Four predictive models were evaluated:

- Neural network with three outputs and all input variable (ANN_1_).
- Neural network with a single output and all input variable (ANN_2_).
- Neural network with three outputs and selected number of input variable (ANN_3_).
- Neural network with a single output and selected number of input variable (ANN_4_).

### ANNs with all variables in input layer, 25 variables

The first implemented neural network models (ANN_1_ and ANN_2_) were developed with all the variables available for tomato samples (Table 1). In this sense, two types of prediction models were studied, first ANN_1_ group with a three output variables, that is, a Neural Network to predict simultaneously the cultivar, the production type and the harvest date, and other group ANN_2_ with three individual neural networks to predict each variable. In Table 2 we can see the APS for the best Neural Networks implemented for each type of output variables selection, that is, for groups ANN_1_ and ANN_2_ implemented with 25 input variables.

**Table 2:** Average Percentage of Success (APS) for the training, validation and average phases (mean APPS for training and validation phase together) considering all variables for harvest date (APS_H_), production type (APS_P_) and the tomato cultivar (APS_C_) for models with 25 input variables (ANN_1_ and ANN_2_) and models with 10 input variables (ANN_3_ and ANN_4_).

			Training phase			Validation phase			Average		
Type	Topology^a^	Training Cycles	APS_H_	APS_P_	APS_C_	APS_H_	APS_P_	APS_C_	APS_H_	APS_P_	APS_C_
ANN_1_	25-41-3	50,000	100	99.3	96.7	68.8	75	50	97	97	92.2
	25-50-3	50,000	100	98.7	95.4	87.5	62.5	12.5	98.8	95.2	87.4
	25-35-3	50,000	99.3	100	94.7	87.5	75	37.5	98.2	97.6	89.2
ANN_2_	25-13-1	2,000	99.3			100			99.4		
	25-28-1	2,000		100			87.5			98.8	
	25-44-1	25,000			99.3			50			94.6
ANN_3_	10-13-3	200,000	87.4	90.1	82.8	68.8	68.8	43.8^b^	85.6	88	79
	10-10-3	200,000	88.7	85.4	64.9	93.8	56.3	31.3	89.2	82.6	61.7
	10-9-3	400,000	68.2	84.1	46.4	75	93.8^b^	18.8	68.9	85	43.7
ANN_4_	10-18-1	1,000	98.7			100^b^			98.8		
	10-8-1	64,000		93.4			81.3			92.2	
	10-18-1	32,000			61.6			31.3			58.7

^a^ The first value corresponds to input variables, the second value corresponds with intermediate neurons, and the last value corresponds with the neurons number in the output layer

^b^ Best models development for the particular case.

As expected, the fits for individual Artificial Neural Network (ANN_2_) were better than the fits for three output variables group (ANN_1_). In Table 2 we can see that all ANN_2_ produce better results for the training phase, except for the Harvest date prediction where the ANN_1_ corresponding to the topologies 25-41-3 and 25-50-3 presented a better adjustment (100%).

The goal of this study is to implement a tool, based on Artificial Neural Networks, to predict the tomato cultivar, production type and harvest date of tomatoes. The choice of the best ANN should not be based on the higher APS in the training phase but in the best APS for validation phase. In the validation phase, all the output values are assumed unknown. Those unknown values are found with the program and later compared with the measured values. This procedure gives us a good idea of the prediction power of the different models to future cases. As we can see in Table 2, the individual model ANN_2_ produces the best prediction results. In this sense, we can see that the individual networks to predict production type and harvest date provide good results, 87.5% and 100% respectively.

A different case is the tomato cultivar. One can see that all models developed presents fits below the fit for production type and harvest date. This behaviour suggests that the 25 nutritional compounds (listed in Table 1) are not suitable for cultivar identification. The inclusion of other compounds is needed for a good prediction of the tomato cultivar.

### ANNs with selected variables in input layer, 10 variables

Due to the good results obtained with all available variables, new ANN models were developed in order to decrease the number of variables in the input layer. These new ANNs are based on the need to reduce personal costs, material and analytical cost, and time. If ANN modelling is used to detect food fraud, those savings can be important. The ANN_3_ models, with three output variables simultaneously, and ANN_4_ models, with one output variable, were developed with 10 input variables of all variables available for tomato samples (Table 2).

The fits for three output Artificial Neural Networks (ANN_3_) are, in general, better than the fits for individual output Neural Networks (ANN_4_). In Table 2 we can see that all ANN_4_ have improved for the training phase, except for the cultivar prediction where the three output ANN_3_ (topology 10-13-3) present a better adjustment than the single ANN_4_ (10-18-1), 82.8% and 61.6% respectively. Contrary to our expectations, the best ANN, in validation phase, for prediction of the tomato cultivar and production type were the ANN_3_ with three output, 10-13-3 (43.8%) and 10-9-3 (93.8%), respectively, and the best ANN_4_ to predict the Harvest date is the individual ANN 10-18-1.

The importance of the variables vary, as expected, in function of ANN model selected. For ANN_4_ (10-18-1), designed to determine the Harvest date, the more important input variables were Lycopene (12.86%), Caffeic (11.89%) and P (11.22%). In the model ANN_3_ (10-9-3) to determine the Production type, the most influential variables were P (16.97%), Caffeic (13.70%) and Na (12.89). Finally, the third model selected, ANN_3_ (10-13-3), to predict the Cultivar, the most important input variables were Cholorogenic (15.25%), Mg (12.23%) and Fe (12.03%)

If we compare the predictions of Table 2 we can see that, as expected, we lost predictive power for tomato cultivar (ANN_1_ 25-41-4 and ANN_2_ 25-44-1 with 50% to ANN_3_ 10-13-3 with 43.8%). However, we gain in predictive power for variable production type (ANN_2_ 25-28-1 with 87.5% to ANN_3_ 10-9-3 with 93.8%) and for harvest date (100%).

Since the aim of this study is to develop a method to detect food fraud by an economical way, quickly and easily, we propose the implementation of a prediction model based only in 10 input variables.

Individual output models are chosen when they offer good individual results, while the multiple outputs models are chosen in function of the average results. However, in this study we propose the model development that use for each output variable the best prediction for the specific output: the model that provides the outputs cultivar and production type, corresponding to the ANN_3_ type (10-13-3 and 10-9-3) and the output of the individual network ANN_4_ (10-18-1) that provides Harvest date.

**Fig 3:**
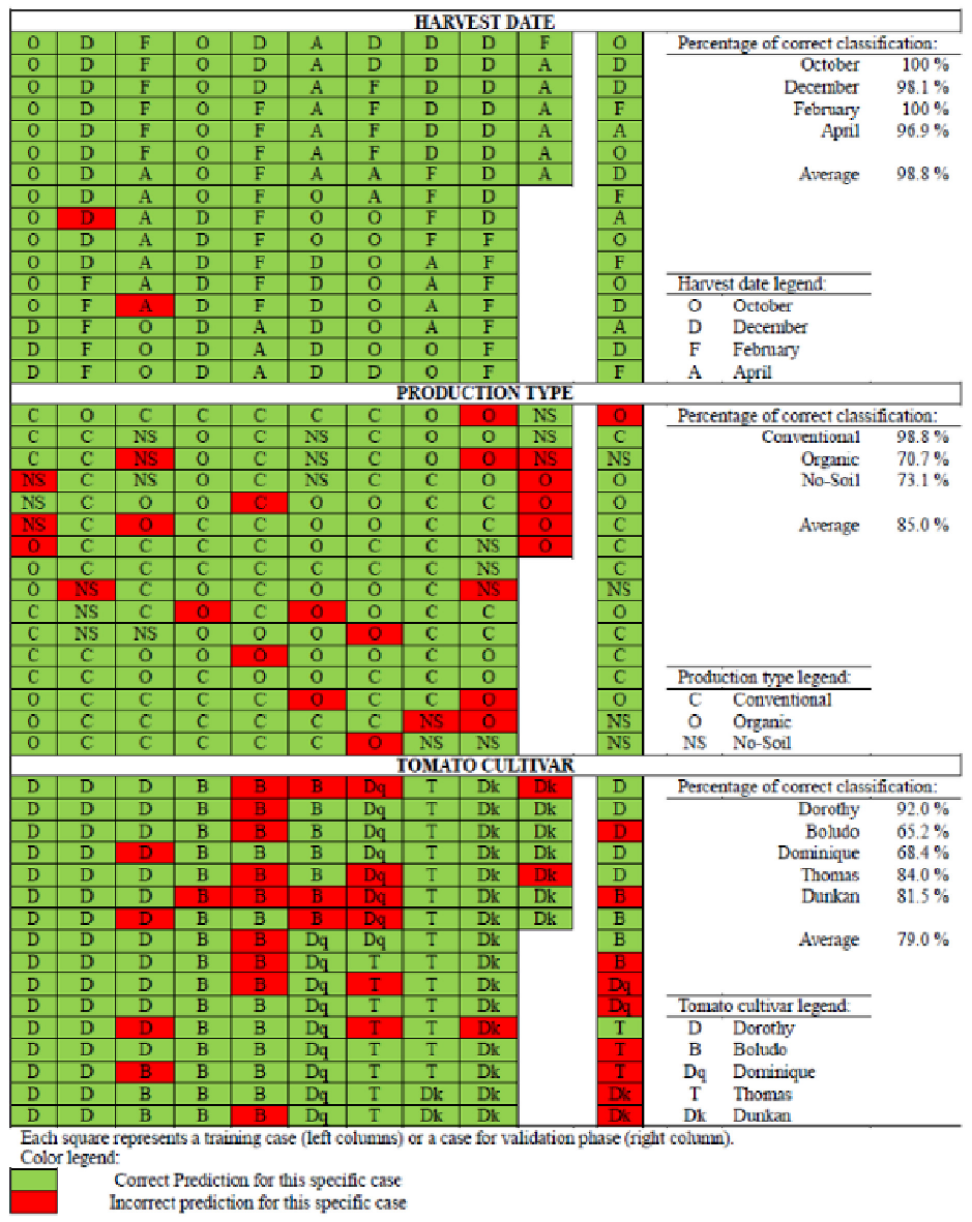
Individual classification for each factor according to prediction models selected to predict the harvests date (D) ANN_4_ 10-18-1, the production type (P) ANN_3_ 10-9-3, and the tomato cultivar (C) ANN_3_ 10-13-3. The left block (ten columns) shows the samples for the training phase (151 cases) and the right block (one column) are the samples for the validation phase (16 cases). The cases correctly classified are highlighted in green whilst the incorrectly classified are in red.

Fig 3 shows the results obtained by the neural networks developed for each output variables, harvest date, production type, and tomato cultivar. This table is divided into three zones, a first zone (upper zone) represents the results for the neural network that predicts the harvest date, a second zone (intermediate zone) for the production type variable, and finally, a third zone (lower zone) for variable cultivar. Each zone has different colored squares that represent specific cases used in the neural network. The squares grouped in the first ten columns on the left are the cases used for the training phase, and the sixteen cases located in the right column are the cases used to validate the networks developed. Inside each square, we can see the variable status (show legend) for each of the cases studied in this paper. Green squares show each of the cases in which the neural model predicts right the output variable (in training or validation phase), while, red squares show a bad prediction of neural model (that’s error prediction). We can see that for the harvest date the model ANN_4_ (10-18-1), offers good results in training (98.7% success, Table 2) and validation phase (100% success, Table 2), likewise, we can see that in the training phase the model makes two errors (Fig 3), one of them for a tomato picked in December (D) and another for tomato harvested in April (A). If we look at the percentage of correct classification for each of the four seasons harvest, for training and validation phase, we can see that the success for October and February seasons are 100%, while for December and April seasons is 98.1 and 96.9%, respectively (Fig 3). On production type variable, prediction model ANN_3_ (10-9-3) offers good results in training (84.1% success, Table 2) and validation phase (93.8% success, Table 2). The only classification error committed in the validation phase belongs to organic production type (O) whose group offers the less percentage of correct classification (70.7%, Fig 3) in the data set to study. Finally, we can see the prediction model ANN_3_ (10-13-3) for cultivar output, this is the group with the worst results for training (82.8%, Table 2) and validation phase (43.8%, Table 2). We can see that in general all cultivars have a good percentage of correct classification, more than 80%, except in cases of Dominique (Dq) and Boludo (B) present 68.4 and 65.2%, respectively (Fig 3). It is notable the behaviour of Dominique (two cases) and Dunkan cultivar (other two cases) in the validation phase, where we can see how the select predictive model is not able to correctly characterize them (Fig 3).

### Comparison of the models

GLM-AID allows a completely separation of tomato samples according to the harvest date (100%) in the first level of the tree-structure. This result agrees with the results for harvest date obtained with the neuronal model developed ANN_4_ (10-18-1), 98.7% and 100% of APS for training and validation phases, 98.8% average. At each collection date, the GLM-AID method produced the right classification of tomatoes according to its production system. However, a complete distinction between the tomato cultivars was not possible with this method. In this case (production system), the prediction model ANN_3_ (10-9-3) provides good adjustment, 84.1% for training phase and 93.8% for validation phase. However, in GLM-AID method, the separation of the cultivar tomato samples was not possible. Similarly, the results with the model prediction model ANN_3_ (10-13-3) give 82.8% for training phase and 43.8% for validation phase. The obtained result can be extrapolated to others harvest seasons, however, we recommended improve these present models with new harvest seasons and other growing areas to improve their prediction before their use around the world. Even models can be improved using local variables such as; climate variables, geographical variables, etc., that can be affect the nutrient content of tomato cultivars.

Regarding the tomato cultivars, they shown a low percentage of classification. Some authors [39, 40] suggest a simultaneous analysis of genetic diversity with various molecular markers in order to obtain a higher efficiency in the levels of genetic variability estimation due to the correlation between data similarity matrices from molecular markers and morphoagronomic. Similarly, our proposed model can be improved by other methods, which would contribute to greater reability to the results obtained.

## Conclusions

Both methods developed, GLM-AID and ANN, have selected different nutritional parameters. The AID analysis does not provide a function that measures the relationship between the dependent variable and the independent variable. However, the AID analysis can be used with other statistical techniques to complete the analysis and determine the relative importance of the different independent variables. In comparison, the ANN creates a function and certain relationships among variables that are more affective in the approximation to each output variable allowing a prediction, more or less accurate, for each output studied variable.

GLM-AID has identified the most significant chemical parameters linked to the harvest date and the production system, and even associated to same tomato cultivars, which can be considered as the main significant differences among tomato samples.

GLM-AID showed that weather conditions have the main influence on the chemical composition of tomatoes followed by the effect of the production system. Once identified those chemical parameters, the tomato cultivars were not sequentially segmented. It can be due to a similar nutritional composition of the tomatoes cultivars.

The ANN models showed different results, where the percentage of classification according to harvest date was 100%, slightly reduced for the production system with 93.8%, and finally the lowest percentage, 50%, for the tomato cultivar. One possible explanation is that the nutritional parameters are not suitable for the identification or classification of tomato cultivars so other kind of markers should be considered. Nevertheless, the combination of GLM-AID and ANN models can be useful to reduce experimental time and cost, personal costs, allowing the use of this predictive models in food fraud detection.

## Supporting Information

S1 Table Mean values according to the harvest date. (DOCX)

S2 Table Mean values according to the agricultural practices. (DOCX)

S3 Table Mean values according to the tomato cultivars. (DOCX)

S4 Table Results of ANNs model. (ZIP)
