# Correction: Identification of Relevant Phytochemical Constituents for Characterization and Authentication of Tomatoes by General Linear Model Linked to Automatic Interaction Detection (GLM-AID) and Artificial Neural Network Models (ANNs)

**DOI:** 10.1371/journal.pone.0134313

**Published:** 2015-07-28

**Authors:** The PLOS ONE Staff

## Notice of Republication

This article was republished on July 9, 2015, to replace incorrect figures. The publisher apologizes for the error. Please download this article again to view the correct version.
