# Supplementary material for: Identification of Relevant Phytochemical Constituents for Characterization and Authentication of Tomatoes by General Linear Model Linked to Automatic Interaction Detection (GLM-AID) and Artificial Neural Network Models (ANNs)
Source: PLoS One. 2015 Jun 15;10(6):e0128566. doi: 10.1371/journal.pone.0128566 (PMC4467870; doi:10.1371/journal.pone.0128566)
Supplement: S1 Table — (DOCX) [file pone.0128566.s001.docx]

|  | October | | December | | February | | April | |
| --- | --- | --- | --- | --- | --- | --- | --- | --- |
|  | Mean | Std deviation | Mean | Std deviation | Mean | Std deviation | Mean | Std deviation |
| Fructose (%) | 1.15 | 0.31 | 1.34 | 0.44 | 1.17 | 0.47 | 1.48 | 0.33 |
| Glucose (%) | 1.03 | 0.26 | 1.36 | 0.36 | 1.27 | 0.47 | 1.53 | 0.40 |
| Total fibre (%) | 1.66 | 0.40 | 1.75 | 0.61 | 1.85 | 0.58 | 2.09 | 0.51 |
| Protein (%) | 0.84 | 0.13 | 0.79 | 0.14 | 0.79 | 0.14 | 0.76 | 0.17 |
| Phenolic compound (mg/100 g) | 20.38 | 4.49 | 21.37 | 3.96 | 19.71 | 5.37 | 19.72 | 3.21 |
| Lycopene (mg/100 g) | 2.92 | 0.72 | 2.22 | 0.48 | 1.67 | 0.47 | 2.51 | 0.59 |
| P (mg/Kg) | 226.52 | 71.57 | 236.61 | 50.07 | 244.71 | 57.90 | 288.69 | 51.45 |
| Na (mg/Kg) | 67.91 | 48.17 | 81.16 | 42.46 | 117.71 | 71.81 | 109.47 | 81.97 |
| K (mg/Kg) | 2414.58 | 662.78 | 2393.16 | 241.67 | 3050.79 | 367.79 | 2194.55 | 247.37 |
| Ca (mg/Kg) | 56.84 | 15.85 | 68.00 | 13.82 | 69.06 | 17.76 | 77.89 | 23.18 |
| Mg (mg/Kg) | 105.86 | 28.64 | 124.48 | 17.19 | 120.47 | 16.92 | 103.74 | 21.23 |
| Fe (mg/Kg) | 1.86 | 0.57 | 1.84 | 0.40 | 1.88 | 0.51 | 2.23 | 0.59 |
| Cu (mg/Kg) | 0.37 | 0.22 | 0.28 | 0.11 | 0.23 | 0.10 | 0.33 | 0.10 |
| Zn (mg/Kg) | 0.78 | 0.23 | 0.73 | 0.19 | 0.71 | 0.20 | 0.91 | 0.17 |
| Mn (mg/Kg) | 0.60 | 0.20 | 0.59 | 0.12 | 0.55 | 0.11 | 0.69 | 0.36 |
| Ascorbic Acid (mg/100 g) | 13.68 | 5.05 | 15.15 | 3.75 | 15.66 | 4.85 | 17.19 | 3.74 |
| Oxalic acid (mg/100 g) | 28.45 | 8.77 | 24.30 | 8.27 | 22.41 | 9.52 | 28.56 | 9.98 |
| Pyruvic acid (mg/100 g) | 1.75 | 0.67 | 1.33 | 0.58 | 0.98 | 0.65 | 1.44 | 1.05 |
| Malic acid (mg/100 g) | 44.05 | 13.39 | 75.46 | 22.17 | 86.61 | 49.63 | 115.44 | 36.83 |
| Citric acid (mg/100 g) | 308.90 | 99.80 | 405.89 | 108.89 | 338.78 | 149.21 | 343.28 | 97.71 |
| Fumaric acid (mg/100 g) | 2.75 | 1.35 | 2.81 | 0.93 | 2.10 | 0.70 | 3.59 | 1.50 |
| Chlorogenic acid (mg/100 g) | 0.41 | 0.22 | 0.45 | 0.32 | 0.85 | 0.62 | 0.73 | 0.43 |
| Caffeic acid (mg/100 g) | 0.05 | 0.02 | 0.03 | 0.02 | 0.03 | 0.01 | 0.03 | 0.01 |
| p-Coumaric acid (mg /100 g) | 0.52 | 0.23 | 0.17 | 0.17 | 0.02 | 0.01 | nd |  |
| Ferulic acid (mg /100 g) | 0.12 | 0.04 | 0.10 | 0.03 | 0.07 | 0.03 | 0.09 | 0.03 |
