# Supplementary material for: Identification of Relevant Phytochemical Constituents for Characterization and Authentication of Tomatoes by General Linear Model Linked to Automatic Interaction Detection (GLM-AID) and Artificial Neural Network Models (ANNs)
Source: PLoS One. 2015 Jun 15;10(6):e0128566. doi: 10.1371/journal.pone.0128566 (PMC4467870; doi:10.1371/journal.pone.0128566)
Supplement: S2 Table — (DOCX) [file pone.0128566.s002.docx]

|  | Conventional | | Organic | | No-soil | |
| --- | --- | --- | --- | --- | --- | --- |
|  | Mean | Std deviation | Mean | Std deviation | Mean | Std deviation |
| Fructose (%) | 1.39 | 0.42 | 1.2 | 0.41 | 1.11 | 0.31 |
| Glucose (%) | 1.41 | 0.41 | 1.21 | 0.4 | 1.1 | 0.31 |
| Total fibre (%) | 1.66 | 0.48 | 1.93 | 0.56 | 2.07 | 0.64 |
| Protein (%) | 0.82 | 0.14 | 0.8 | 0.15 | 0.72 | 0.14 |
| Phenolic compound (mg/100 g) | 20.46 | 4.96 | 20.9 | 3.84 | 19.15 | 3.22 |
| Lycopene (mg/100 g) | 2.32 | 0.71 | 2.38 | 0.77 | 2.11 | 0.58 |
| P (mg/Kg) | 228.04 | 61.06 | 249.39 | 53.89 | 296.81 | 49.52 |
| Na (mg/Kg) | 114.22 | 64.23 | 84.65 | 59.86 | 39.91 | 19.87 |
| K (mg/Kg) | 2494.52 | 509.43 | 2614.02 | 532.82 | 2405.5 | 457.34 |
| Ca (mg/Kg) | 63.83 | 16.27 | 66.67 | 17.47 | 80.94 | 22.23 |
| Mg (mg/Kg) | 117.17 | 20.68 | 121.26 | 21.54 | 94.93 | 21.39 |
| Fe (mg/Kg) | 1.88 | 0.55 | 2.00 | 0.46 | 1.93 | 0.6 |
| Cu (mg/Kg) | 0.25 | 0.11 | 0.34 | 0.12 | 0.36 | 0.24 |
| Zn (mg/Kg) | 0.7 | 0.22 | 0.83 | 0.17 | 0.87 | 0.17 |
| Mn (mg/Kg) | 0.57 | 0.15 | 0.57 | 0.12 | 0.78 | 0.37 |
| Ascorbic Acid (mg/100 g) | 15.61 | 4.84 | 15.48 | 4.01 | 13.96 | 4.21 |
| Oxalic acid (mg/100 g) | 28.06 | 10.1 | 23.09 | 8.27 | 23.65 | 7.01 |
| Pyruvic acid (mg/100 g) | 1.37 | 0.74 | 1.41 | 0.88 | 1.23 | 0.59 |
| Malic acid (mg/100 g) | 85 | 44.71 | 70.79 | 37.25 | 73.88 | 26.41 |
| Citric acid (mg/100 g) | 381.71 | 128.85 | 331.45 | 118.55 | 317.04 | 78.02 |
| Fumaric acid (mg/100 g) | 2.82 | 1.29 | 2.67 | 1.18 | 2.85 | 1.12 |
| Chlorogenic acid (mg/100 g) | 0.63 | 0.49 | 0.58 | 0.43 | 0.53 | 0.38 |
| Caffeic acid (mg/100 g) | 0.03 | 0.02 | 0.04 | 0.02 | 0.03 | 0.02 |
| p-Coumaric acid (mg /100 g) | 0.02 | 0.02 | 0.02 | 0.03 | 0.01 | 0.02 |
| Ferulic acid (mg /100 g) | 0.1 | 0.04 | 0.09 | 0.04 | 0.08 | 0.03 |
