# Supplementary material for: Identification of Relevant Phytochemical Constituents for Characterization and Authentication of Tomatoes by General Linear Model Linked to Automatic Interaction Detection (GLM-AID) and Artificial Neural Network Models (ANNs)
Source: PLoS One. 2015 Jun 15;10(6):e0128566. doi: 10.1371/journal.pone.0128566 (PMC4467870; doi:10.1371/journal.pone.0128566)
Supplement: S3 Table — (DOCX) [file pone.0128566.s003.docx]

|  | Dorothy | | Boludo | | Dominique | | Thomas | | Dunkan | |
| --- | --- | --- | --- | --- | --- | --- | --- | --- | --- | --- |
|  | Mean | Std deviation | Mean | Std deviation | Mean | Std deviation | Mean | Std deviation | Mean | Std deviation |
| Fructose (%) | 1.20 | 0.30 | 1.35 | 0.39 | 1.50 | 0.54 | 1.26 | 0.47 | 1.19 | 0.44 |
| Glucose (%) | 1.18 | 0.31 | 1.40 | 0.37 | 1.50 | 0.52 | 1.30 | 0.49 | 1.17 | 0.40 |
| Total fibre (%) | 1.88 | 0.49 | 1.84 | 0.49 | 1.84 | 0.80 | 1.74 | 0.66 | 1.71 | 0.48 |
| Protein (%) | 0.79 | 0.16 | 0.78 | 0.13 | 0.86 | 0.16 | 0.82 | 0.12 | 0.78 | 0.16 |
| Phenolic compound (mg/100 g) | 21.18 | 4.42 | 20.30 | 4.36 | 20.42 | 4.85 | 19.56 | 5.10 | 19.93 | 3.19 |
| Lycopene (mg/100 g) | 2.31 | 0.71 | 2.44 | 0.77 | 2.27 | 0.83 | 2.55 | 0.65 | 1.89 | 0.44 |
| P (mg/Kg) | 234.56 | 55.49 | 253.69 | 58.59 | 231.79 | 73.31 | 248.85 | 65.03 | 262.43 | 62.91 |
| Na (mg/Kg) | 87.48 | 68.04 | 86.95 | 65.29 | 87.46 | 49.71 | 93.53 | 32.29 | 113.11 | 79.47 |
| K (mg/Kg) | 2482.47 | 546.86 | 2565.08 | 545.56 | 2668.32 | 617.27 | 2485.04 | 384.78 | 2453.66 | 413.67 |
| Ca (mg/Kg) | 70.05 | 18.74 | 66.70 | 23.68 | 57.51 | 15.24 | 69.31 | 13.70 | 69.37 | 12.01 |
| Mg (mg/Kg) | 110.90 | 20.38 | 116.60 | 22.10 | 122.70 | 30.45 | 114.93 | 26.00 | 115.07 | 18.34 |
| Fe (mg/Kg) | 1.77 | 0.52 | 1.98 | 0.46 | 1.84 | 0.43 | 1.89 | 0.44 | 2.23 | 0.68 |
| Cu (mg/Kg) | 0.32 | 0.13 | 0.28 | 0.13 | 0.27 | 0.13 | 0.29 | 0.11 | 0.32 | 0.24 |
| Zn (mg/Kg) | 0.75 | 0.22 | 0.79 | 0.20 | 0.77 | 0.24 | 0.81 | 0.21 | 0.75 | 0.18 |
| Mn (mg/Kg) | 0.59 | 0.16 | 0.63 | 0.31 | 0.62 | 0.12 | 0.61 | 0.15 | 0.55 | 0.17 |
| Ascorbic Acid (mg/100 g) | 15.14 | 4.30 | 15.78 | 5.24 | 14.26 | 4.33 | 16.04 | 4.61 | 14.89 | 3.37 |
| Oxalic acid (mg/100 g) | 25.2 | 10.20 | 25.67 | 8.39 | 24.89 | 8.89 | 29.32 | 9.30 | 23.56 | 9.21 |
| Pyruvic acid (mg/100 g) | 1.22 | 0.76 | 1.22 | 0.59 | 1.53 | 0.77 | 1.64 | 0.57 | 1.52 | 1.09 |
| Malic acid (mg/100 g) | 71.44 | 31.93 | 89.7 | 37.35 | 68.21 | 32.83 | 66.28 | 34.53 | 90.04 | 58.71 |
| Citric acid (mg/100 g) | 340.08 | 104.94 | 389.24 | 130.07 | 353.32 | 142.66 | 321.6 | 110.04 | 351.37 | 122.83 |
| Fumaric acid (mg/100 g) | 3.04 | 1.44 | 2.85 | 1.19 | 2.63 | 1.21 | 2.32 | 0.99 | 2.67 | 0.92 |
| Chlorogenic acid (mg/100 g) | 0.54 | 0.46 | 0.75 | 0.54 | 0.67 | 0.41 | 0.49 | 0.26 | 0.47 | 0.38 |
| Caffeic acid (mg/100 g) | 0.03 | 0.02 | 0.03 | 0.02 | 0.04 | 0.01 | 0.04 | 0.02 | 0.03 | 0.02 |
| p-Coumaric acid (mg /100 g) | 0.02 | 0.03 | 0.01 | 0.02 | 0.02 | 0.03 | 0.02 | 0.03 | 0.01 | 0.02 |
| Ferulic acid (mg /100 g) | 0.11 | 0.04 | 0.09 | 0.03 | 0.09 | 0.03 | 0.11 | 0.03 | 0.07 | 0.04 |
